# Defining the phenotype of neutrophils following reverse migration in zebrafish

**DOI:** 10.1189/jlb.3MA0315-105R

**Published:** 2015-06-12

**Authors:** Felix Ellett, Philip M. Elks, Anne L. Robertson, Nikolay V. Ogryzko, Stephen A. Renshaw

**Affiliations:** Bateson Centre and Department of Infection and Immunity, University of Sheffield, Sheffield, United Kingdom

**Keywords:** granulocytes, inflammation, chemotaxis, infection, phagocytosis

## Abstract

Stimulation of neutrophil reverse migration presents an attractive, alternative therapeutic pathway to driving inflammation resolution. However, little is known about whether the activity of wound-experienced neutrophils is altered and whether encouraging dispersal of such neutrophils back into the body may have undesirable consequences. This study used a zebrafish tail transection inflammation model, in combination with a photoconvertible neutrophil transgenic line, to allow internally controlled, simultaneous comparison of reverse-migrated neutrophils with naïve neutrophils in the presence and absence of secondary insult. Detailed microscopy revealed that reverse-migrated neutrophils exhibited an activated morphology but responded normally to secondary insult and are able to mount an effective antimicrobial response to *Staphylococcus aureus*. These results support a model in which reverse-migrated neutrophils exhibit no long-term behavioral alterations and encourage the notion of enhanced reverse migration as a viable target for pharmaceutical manipulation.

## Abstract (precis)

Zebrafish reverse-migrated neutrophils retain the ability to migrate to secondary wounds and mount a robust antibacterial response.

## Introduction

Failure of inflammation resolution underlies the pathology of many chronic diseases, including atherosclerosis, rheumatoid arthritis, osteoporosis, and chronic obstructive pulmonary disease [1]. Neutrophils aberrantly retained at sites of injury can cause collateral damage to surrounding tissue [2, 3], stimulating recruitment of additional inflammatory cells and perpetuating an inflammatory environment [4, 5]. Macrophages have a well-described role in removal of neutrophils from sites of inflammation by efferocytosis of apoptotic neutrophils to limit off-target damage [6]. An additional pathway for removal of neutrophils by reverse migration away from inflammatory sites has been described more recently [7–10] and provides an attractive therapeutic pathway to remove neutrophils.

We have shown previously that neutrophil reverse migration is regulated by proinflammatory stimuli, such as hypoxia [8], and can be accelerated by potential proresolution therapeutics [11]. Other groups have suggested that initiation of reverse migration is dependent on the arrival of macrophages at the wound and is likely to be mediated by a combination of direct contact inhibition [12] and production of diffusible proresolution compounds. Mathematical modeling of neutrophil behavior during inflammation resolution in zebrafish larvae indicates that neutrophils leave the wound in a stochastic manner akin to diffusion, suggesting loss of sensitivity toward recruitment and/or retention signal(s) rather than active repulsion (fugetaxis) [13]. However, driving the active redistribution of wound-experienced neutrophils throughout the body could have potentially unwanted side effects. Human neutrophils exhibiting a reverse-transmigrated phenotype displayed delayed apoptosis and increased ROS production [7], whereas studies in murine systems have suggested that neutrophils activated during ischemia-reperfusion injury may be primed to initiate inflammation elsewhere following reverse transmigration back into the circulation [10]. Conversely, it is possible that neutrophils might be defective in targeting to other stimuli or in bacterial killing following activation at wounds.

In the zebrafish model, previous studies of neutrophil trafficking following injury did not identify any pattern to neutrophil dispersal following reverse migration [14, 15]. Therefore, a key question is whether neutrophils that have participated in the inflammatory response carry some other memory of this experience. In this study, we focused specifically on whether the wound experience has a detectable effect on neutrophil phenotype following reverse migration, including assessment of whether reverse-migrated neutrophils were able to mount a robust response to secondary insults.

## MATERIALS AND METHODS

### Zebrafish

Adult zebrafish were maintained according to standard procedures [16]. *Tg(mpx:Gal4/UAS:Kaede)* embryos were generated by in-crossing *Tg(mpx:Gal4.VP16)^i222^;Tg(UAS:Kaede)^s1999t^* [11] adults. For all experiments, embryo tails were transected distally to the tip of the notochord at 48 h postfertilization. Kaede-expressing wound neutrophils were photoconverted at 6 hpw, as described previously [13, 17]. All further assays and imaging were carried out 24 hpw.

### *S. aureus* infection models

*S. aureus* strain SH1000 [18] was used for all experiments. Strains expressing enhanced GFP and mCherry were generated by transformation with an appropriate tetracycline-resistance plasmid. Staining of *S. aureus* with pHrodo and Alexa Fluor 647 (Life Technologies, Carlsbad, CA, USA) was carried out as follows: pelleted *S. aureus* were resuspended to 2.5 × 10^3^ CFU/nl in 200 µl PBS (pH 9), 0.5 µl (pHrodo), and/or 5 µl (Alexa Fluor 647) dye (10 mg/ml) was added and the tube shaken for 30 min at 37°C. To wash, bacteria were then pelleted (16.3 *g* for 3 min) and resuspended sequentially in 1 ml PBS (pH 8), 1 ml 25 mM Tris (pH 8.5), and 1 ml PBS (pH 8) before final resuspension in 200 µl PBS (pH 7.4). Costaining with CellROX (Life Technologies) was performed by adding 0.5 µl dye (10 mg/ml) to 200 µl bacteria and coincubating as described above. Bacteria were then pelleted and washed twice in 1 ml PBS (pH 7.4) before resuspension in 200 µl.

### Microscopy

Widefield, time-lapse microscopy was performed by use of a Nikon Eclipse TE2000-U inverted compound fluorescence microscope fitted with a 10×, 0.30 NA objective and a 1394 ORCA-ERA camera (Hamamatsu Photonics, Hamamatsu City, Japan).

High-resolution imaging and Kaede photoconversion were performed by use of an inverted UltraVIEW VoX spinning disk confocal microscope (PerkinElmer Life and Analytical Sciences, Shelton, CT, USA), fitted with a 40×, 0.76 NA oil-immersion objective.

### Analysis and statistics

Volumetric and shape-factor analyses were performed by use of Volocity 6.3 (PerkinElmer Life and Analytical Sciences) software by use of intensity of fluorescence to identify individual cells. Cell tracking was performed manually.

Graphing and statistical analysis was performed by use of Prism Version 6.0c (GraphPad Software, La Jolla, CA, USA). Paired *t* tests were used to compare data for cells within the same field of view or for nearest-neighbor analysis of tracking data.

## RESULTS

### An internally controlled model for comparison of reverse-migrated and naïve neutrophils

The zebrafish tail transection model, combined with photoconvertible neutrophil-specific transgenic lines, has provided many insights into mechanisms affecting neutrophil-reverse migration [8, 14]. It also provides a unique opportunity to compare directly the behavior of photoconverted reverse-migrated neutrophils with naïve neutrophils in the presence and absence of a secondary insult.

**Figure 1.**
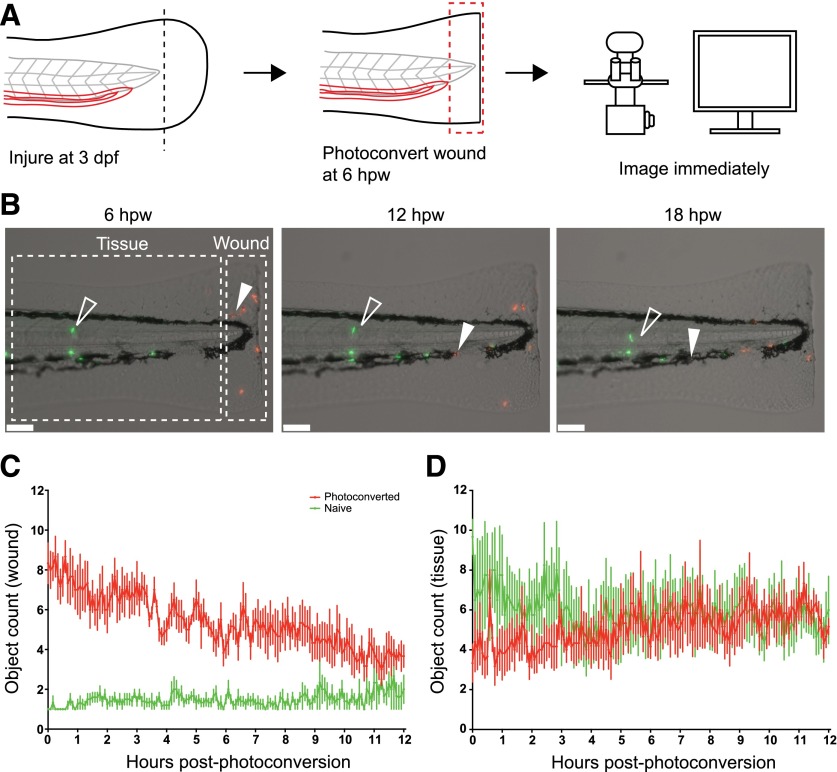
Identifying reverse-migrated neutrophils following tail transection. (A) Experimental approach. Tails of 3 d postfertilization (dpf) *Tg(mpx:Gal4/UAS:Kaede)* were transected caudally to the tip of the notochord. Neutrophils at the wound were photoconverted at the peak of inflammation (6 hpw) and larvae imaged for 12 h. (B) Representative images extracted from a 12 h time-lapse movie following photoconversion. Red photoconverted neutrophils reverse migrating back into the tissue (filled, white arrowheads) are easily distinguished from green naïve neutrophils (open, white arrowheads). Scale bars, 100 µm. (C) Object counts at the wound following photoconversion. Most photoconverted neutrophils reverse migrate by 12 h postphotoconversion, whereas few new green neutrophils enter the wound zone. (D) Representative object counts in the tissue. Reverse migration of photoconverted neutrophils results in a gradual increase in object counts in the tissue, whereas green naïve cell numbers decrease slightly as they are mobilized from the caudal hematopoietic tissue into the bloodstream. *n* = 6 embryos, mean ± sem.

For these studies, we chose an intermediate degree of tissue injury, transecting the tailfin distally to the notochord. This injury causes localized tissue damage but does not damage the muscle, notochord, or neural tube. Neutrophil inflammation peaked at ∼6 hpw and was followed by spontaneous inflammation resolution, as described previously [19] (data not shown). We defined the wound region as the 200 µm of tissue proximal to the wound edge (**Fig 1B**). Wound neutrophils were photoconverted at 6 hpw and the larvae imaged for 12 h (Fig. 1A and B and Supplemental Movie 1). Wound neutrophils began to reverse migrate from 6 hpw, with few naïve neutrophils arriving at the wound (Fig. 1C and D).

By 24 hpw, the neutrophilic component of the inflammation had resolved completely, with photoconverted reverse-migrated neutrophils distributed throughout the embryo, as described previously [8, 11, 13, 14]. This provided an ideal situation in which to test whether reverse-migrated neutrophils exhibited any long-term behavioral changes compared with nearby naïve neutrophils. Therefore, subsequent experiments were designed to use naïve neutrophils as an internal control for reverse-migrated neutrophils in the same field of view, which allowed paired comparison, either within the field or to the nearest neighboring cell.

### Reverse-migrated neutrophils display an activated morphology unrelated to basal migration behavior

Neutrophil activation at the site of wounding occurs via multiple pathways, following interaction with damage-associated molecular patterns, pathogen-associated molecular patterns, and host-derived signaling molecules [20–24]. Following priming, neutrophils take on an “activated” morphology [25], exhibiting multiple protrusions and enhanced ROS generation [26, 27].

**Figure 2.**
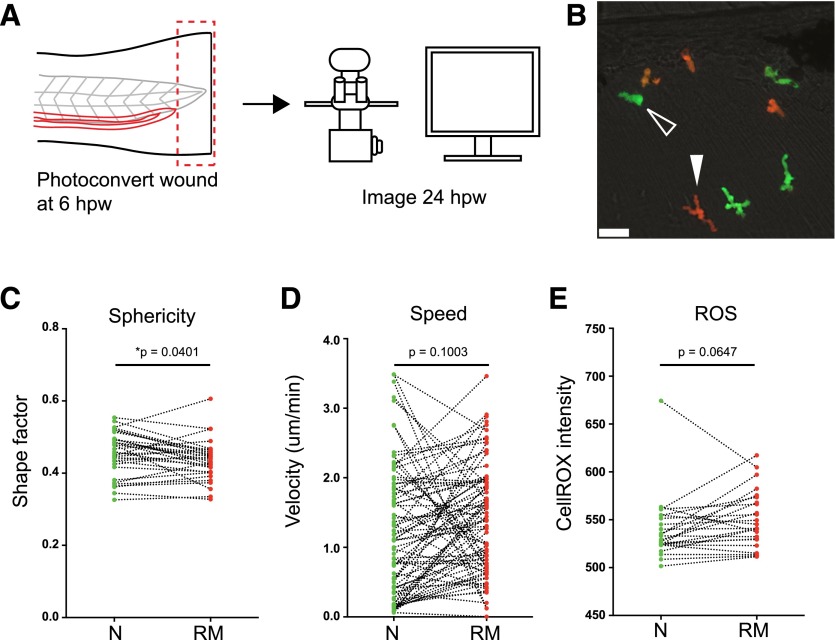
Reverse-migrated neutrophils display activated cell morphology. (A) Experimental approach. Tails of 3 dpf *Tg(mpx:Gal4/UAS:Kaede)* were transected caudally to the tip of the notochord. Neutrophils at the wound were photoconverted at the peak of inflammation (6 hpw). Larvae were then imaged 24 h postinjury by confocal microscopy (B, C, and E) or imaged for 6 h by use of a widefield microscope (D). (B) Representative image comparing cellular morphology of green naïve neutrophils (open, white arrowhead) with red reverse-migrated neutrophils (filled, white arrowhead) at 24 hpw. Scale bar, 25 µm. (C) Paired comparison of cell shape between naïve (N) and reverse-migrated (RM) neutrophils at 24 hpw. Reverse-migrated neutrophils were significantly less spherical than naïve cells. Data shown are paired average shape factor of naïve and reverse-migrated neutrophils, *n* = 33 fields of view (larvae), *n* = 3 experiments. Paired, two-tailed *t* test. (D) Paired (nearest-neighbor) comparison of cell migration of naïve and reverse-migrated neutrophils from manual cell-tracking data at 24 hpw. *n* = 78 paired cells from 9 larvae, *n* = 3 experiments. Paired, two-tailed *t* test. (E) Paired comparison of ROS between naïve and reverse-migrated neutrophils at 24 hpw. Data shown are paired average CellROX fluorescence intensity of naïve and reverse-migrated neutrophils, *n* = 26 paired fields of view (larvae), *n* = 3 experiments. Paired, two-tailed *t* test.

To test the hypothesis that reverse-migrated neutrophils may retain an activated phenotype, the shape factor (sphericity) of reverse-migrated neutrophils was compared with nearby naïve neutrophils (**Fig. 2A–C**) [25]. Reverse-migrated neutrophils displayed a significantly lower shape factor compared with naïve neutrophils, suggesting that they may be activated [25]. This effect was not a result of photoconversion, as comparison of photoconversion of neutrophils in unwounded fish did not lead to a change in shape factor compared with unconverted neutrophils (Supplemental Fig. 1A–H). Likewise, reverse-migrated neutrophils identified by tracking at earlier time-points had indistinguishable velocity profiles to photoconverted cells nearby (Supplemental Fig. 1I–K).

Neutrophil shape varies greatly depending on the surrounding tissue and whether the cell is actively migrating. The activated morphology observed in reverse-migrated cells suggested that these cells might exhibit modified migration properties. To assess basal migration rates, reverse-migrated and naïve neutrophils were tracked in the absence of a secondary insult and nearest-neighbor comparisons performed. Reverse-migrated neutrophils displayed comparative velocity with naïve cells (Fig. 2D), suggesting that the morphologic differences observed were not a result of different migration behavior.

If the observed increase in shape factor does indicate increased activation or priming, this might be associated with increased production of ROS. To test whether reverse-migrated neutrophils showed increased ROS generation, embryos were immersed in CellROX, a cell-permeable, fluorescent indicator of oxidative stress, and imaged. No significant difference in CellROX fluorescence intensity was observed in reverse-migrated neutrophils compared with naïve neutrophils in the absence of a secondary insult (Fig. 2E).

### Reverse-migrated neutrophils respond normally to sterile and nonsterile secondary insult

**Figure 3.**
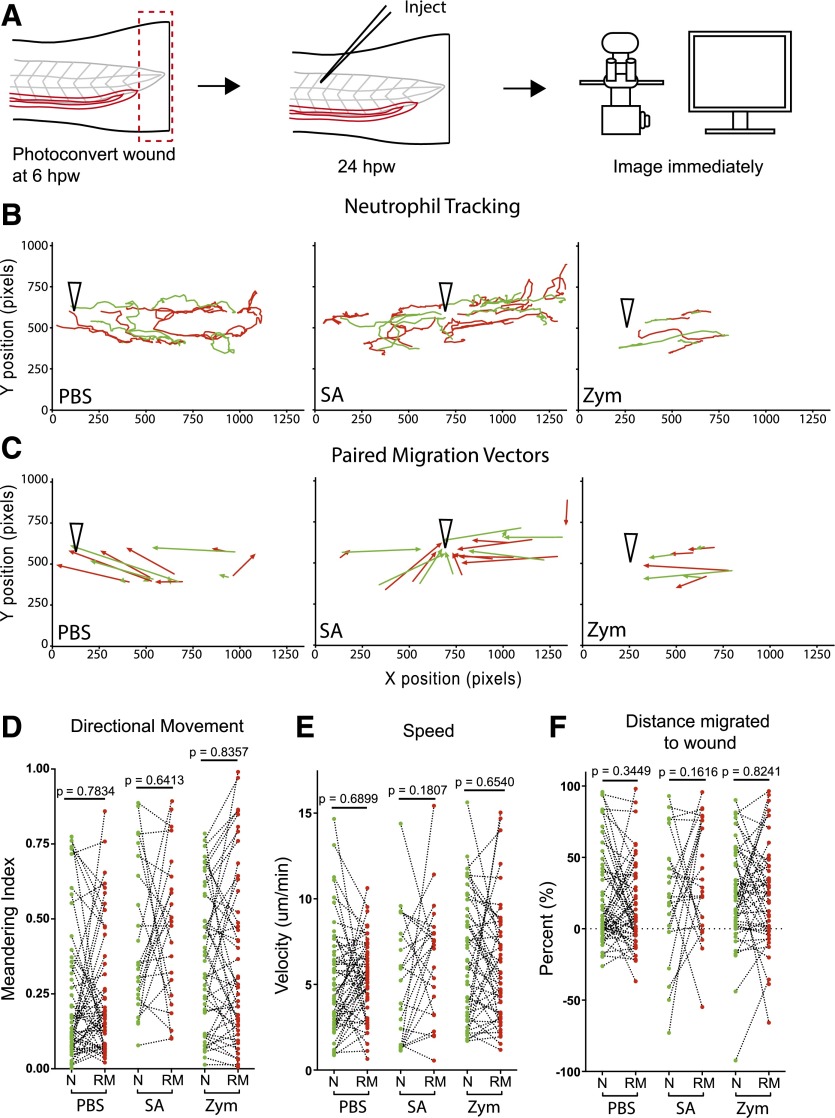
Reverse-migrated neutrophils migrate normally to secondary insult. (A) Experimental approach. Tails of 3 dpf *Tg(mpx:Gal4/UAS:Kaede)* were transected caudally to the tip of the notochord. Neutrophils at the wound were photoconverted at the peak of inflammation (6 hpw). At 24 hpw, larvae were injected with PBS, GFP +ve *S. aureus*, or red fluorescent zymosan into the somite and imaged immediately on a widefield microscope for 6 h. (B) Representative paired (nearest-neighbor) tracks for naïve (green lines) and reverse-migrated (red lines) neutrophils toward injection site (open, black arrowheads) for larvae injected with PBS, *S. aureus* (SA), and zymosan (Zym). Robust migration of both naïve and reverse-migrated neutrophils was observed in response to secondary insult. (C) Corresponding migration vectors for tracks shown in B. (D–F) Paired (nearest-neighbor) comparison of naïve and reverse-migrated neutrophil migration in response to secondary insult. No difference in meandering index (D), velocity (E), or percent of initial distance migrated toward the wound (F) was observed between naïve and reverse-migrated neutrophils in response to PBS, *S. aureus*, or zymosan injection. For PBS, *n* = 51 cell pairs from 9 larvae, *n* = 3 experiments. For *S. aureus*, *n* = 23 cell pairs from 9 larvae, *n* = 3 experiments. For zymosan, *n* = 46 cell pairs from 12 larvae, *n* = 3 experiments. Paired, two-tailed *t* test.

The mechanisms by which neutrophils respond differently to wound cues to initiate reverse migration is not known, but one explanation is that there may be altered sensitivity to chemotactic signals [13, 17]. To test whether reverse-migrated neutrophils respond normally to a secondary insult, larvae were injected with PBS, *S. aureus*, or zymosan into the 26–27th somite, 24 h post-tail transection and neutrophils tracked (**Fig. 3A–C** and Supplemental Movies 2 and 3). Nearest-neighbor comparison of reverse-migrated and naïve neutrophils showed no significant difference in directionality, velocity, or migration toward the injection wound (Fig. 3D–F). Thus, reverse-migrated neutrophils are neither primed to respond to, nor desensitized toward, a secondary insult.

### Reverse-migrated neutrophils mount a normal antimicrobial defense

**Figure 4.**
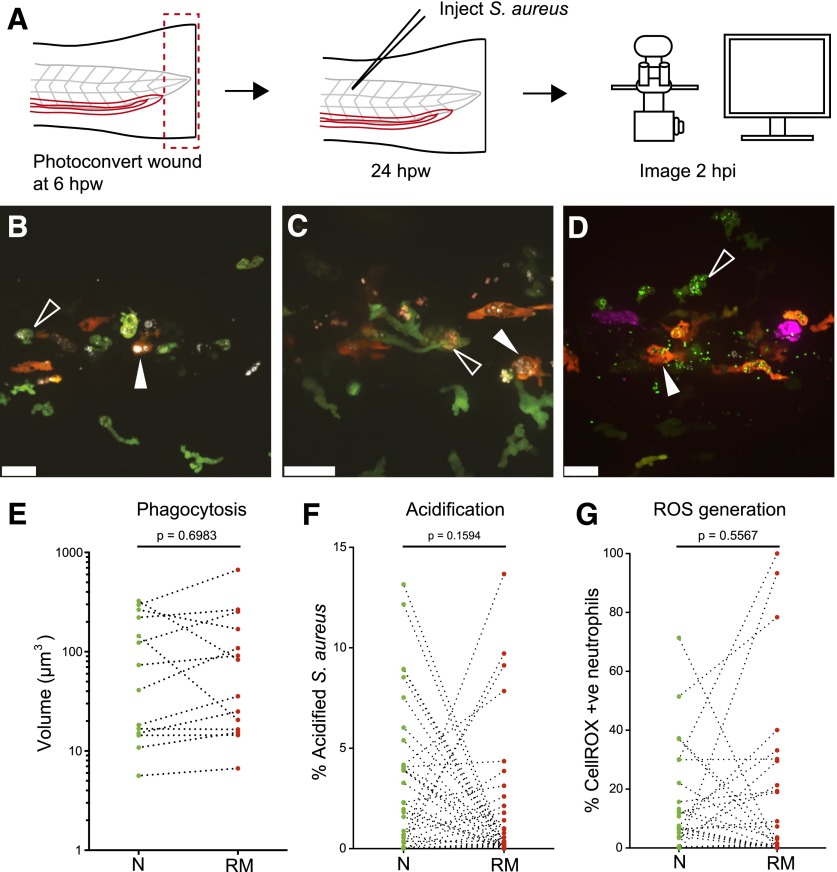
Reverse-migrated neutrophils display unchanged host-defense capacity. (A) Experimental approach. Tails of 3 dpf *Tg(mpx:Gal4/UAS:Kaede)* were transected caudally to the tip of the notochord. Neutrophils at the wound were photoconverted at the peak of inflammation (6 hpw). At 24 hpw, larvae were injected with *S. aureus* and imaged 2 hpi by use of a confocal microscope. (B) Representative image of naïve (green; open, white arrowhead) and reverse-migrated (red; filled, white arrowhead) neutrophils containing phagocytosed *S. aureus* labeled with Alexa Fluor 647 (white) at 2 hpi. Scale bar, 25 µm. (C) Representative image of naïve (green; open, white arrowhead) and reverse-migrated (red; filled, white arrowhead) neutrophils containing phagocytosed *S. aureus* colabeled with Alexa Fluor 647 (white) and pHrodo (red) at 2 hpi. Scale bar, 25 µm. (D) Representative image of naïve (green; open, white arrowhead) and reverse-migrated (red; filled, white arrowhead) neutrophils containing phagocytosed GFP-transgenic *S. aureus* (green) stained with CellROX (magenta) at 2 hpi. Scale bar, 25 µm. (E–G) Paired comparison of host defense for naïve and reverse-migrated neutrophils following infection with *S. aureus*. No difference was observed for volume phagocytosed (E), bacterial acidification (F), and ROS (G). Data shown are paired averages/field of view. For phagocytosis, *n* = 14 fields (larvae), *n* = 3 experiments. For acidification, *n* = 38 fields (larvae), *n* = 4 experiments. For ROS, *n* = 36 fields (larvae), *n* = 4 experiments.

At the wound, neutrophils play an important antimicrobial role by phagocytosing bacteria and killing them in phagolysosomes [28, 29]. The most common bacterial infection following human injury is by the opportunistically pathogenic, commensal *S. aureus* [30]. To test whether reverse-migrated neutrophils display an altered antimicrobial defense, larvae were injected with *S. aureus* into the dorsal segment of the 17th somite (directly above the caudal end of the yolk extension), 24 h post-tailfin transection. Preliminary time-lapse microscopy revealed that phagocytosis and killing of *S. aureus* by neutrophils occurred from 30 min to 3 hpi in this model (data not shown). Therefore, phagocytosis, phagosome pH and bacterial oxidative stress were assessed at 2 hpi by confocal microscopy (**Fig. 4A**) so that enhanced responses could be identified.

To assess phagocytosis, *S. aureus* were prestained with Alexa Fluor 647 (far red emission, shown as white in Fig. 4), and intracellular bacterial volumes were assessed by image quantification, comparing naïve neutrophils (green) and reverse-migrated neutrophils (red) at 2 hpi (Fig, 4B). Volocity imaging software was used to assign volumes based on Alexa Fluor 647 fluorescent intensity (Fig 4E). No differences in amount of phagocytosis by neutrophils were observed, suggesting that phagocytic capacity is not influenced by neutrophil wound experience.

Therefore, we went on to test whether neutrophils were able to process phagocytosed bacteria normally following reverse migration. To investigate phagosome maturation in neutrophils in vivo [31], *S. aureus* were costained with Alexa Fluor 647 and pHrodo, which exhibits increased red fluorescence as pH decreases [32] (Fig. 4C). Stained bacteria were injected into zebrafish muscle following tailfin injury and photoconversion as above. Zebrafish were then imaged, and Volocity imaging software was used to calculate the percentage of intracellular Alexa Fluor 647-positive *S. aureus* that exhibited high pHrodo fluorescence (Fig 4F). No differences were seen in this assay between naïve and reverse-migrated neutrophils.

To measure bacterial oxidative stress, GFP transgenic *S. aureus* were stained with CellROX before infection (Fig. 4D). Therefore, CellROX positivity of *S. aureus*-containing cells was used as an indicator of neutrophil bactericidal activity (Fig. 4G). Paired comparison of reverse-migrated and naïve neutrophils for each field of view revealed no significant differences, suggesting that reverse-migrated neutrophils are able to phagocytose and kill bacteria normally.

## DISCUSSION

Neutrophils must be removed from inflammatory sites for inflammation to resolve. Neutrophil apoptosis leads to down-regulation of neutrophil proinflammatory functions. Clearance of apoptotic neutrophils by macrophages, in turn, leads to down-regulation of macrophage phlogistic potential [33]. It is now becoming accepted that the more recently described phenomenon of reverse migration makes a significant contribution to inflammation resolution in certain contexts [34]. Therefore, a key question is whether this dissipation of the inflammatory burden is associated with any phenotypic alteration in the neutrophils themselves. This is particularly important if we consider therapeutic induction of reverse migration as a potential strategy for treating inflammatory disease.

Our previous work demonstrated that tail transection of zebrafish embryos results in up-regulation of IL-1β throughout the entire embryo by 2 hpw [35], suggesting a widespread activation of leukocytes upon injury. In this study, we specifically addressed the hypothesis that the wound experience and subsequent reverse migration may alter the ability of neutrophils to respond to secondary insult and infection.

The zebrafish reverse-migration model offers the advantage of superior imaging throughout the entire inflammatory time course. This has been exploited for cell tracking and imaging with a range of fluorescent transgenic lines and chemical indicators of neutrophil functional capacity.

To address the hypotheses of this study, a number of new assays were developed. The experiments described were internally controlled, simultaneously improving statistical power and minimizing sample-to-sample variation inherent to neutrophil behavior. This approach also avoided confounding effects, such as generalized activation of neutrophils upon wounding, which might bias observations made between injured and uninjured larvae.

The potential for the imaging assays used to identify differences in neutrophil antimicrobial activity, such as phagosome maturation and ROS generation, is clearly demonstrated by the variation observed within samples. Although differences between reverse-migrated and naïve neutrophils were observed within samples, these were not consistent between samples. This suggests that although antimicrobial activity may vary between neutrophils, that activity is not influenced by wound experience.

The zebrafish larvae provides an excellent model to image host-pathogen interactions in vivo [36]. This is particularly true for neutrophil-pathogen interaction, which is intrinsically difficult to image in in vivo mammalian models and plagued by artifact complications in in vitro systems [37]. Many new transgenic markers of subcellular structures, in combination with indicator dyes, such as those described here, will provide important tools for future assay design.

The assays developed for this study were designed to address important aspects of neutrophil function that might be potentially altered following wound experience and reverse migration. It is clear from the results presented that reverse-migrated neutrophils have no discernible deficiencies in their ability to mount a rapid and effective response to secondary insult and infection. More subtle aspects of neutrophil behavior, such as endothelial transmigration, surface phenotype, and lifespan, have been addressed recently in other experimental systems [7, 38].

In this study, we show that in the context of basal activity, migration to sites of secondary injury and infection, and antimicrobial activity, reverse-migrated neutrophils display unaltered behavior compared with neutrophils that are not wound experienced. The application of these assays to neutrophils in injured versus uninjured larvae to investigate the effects of long-range neutrophil activation on neutrophil phenotype may provide an interesting direction for future studies.

## AUTHORSHIP

F.E. and S.A.R. designed the experiments and wrote the manuscript. F.E. and P.M.E. performed the experiments. A.L.R., N.V.O., and P.M.E. were involved in experimental design and provided advice on the manuscript.

## Abbreviations

hpi: hours postinfection
hpw: hours postwounding
MRC: Medical Research Council
ROS: reactive oxygen species
